# Co-culture of Platelets with Monocytes Induced M2 Macrophage Polarization and Formation of Foam Cells: Shedding Light on the Crucial Role of Platelets in Monocyte Differentiation

**DOI:** 10.4274/tjh.galenos.2019.2018.0449

**Published:** 2019-05-03

**Authors:** Mahdieh Mehrpouri, Davood Bashash, Mohammad Hossien Mohammadi, Mohammad Esmail Gheydari, Esmail Shahabi Satlsar, Mohsen Hamidpour

**Affiliations:** 1 Shahid Beheshti University of Medical Sciences, Faculty of Allied Medical Sciences, Department of Hematology and Blood Banking, Tehran, Iran; 2 HSCT Research Centre, Department of Hematology and Blood Banking, School of Allied Medical Sciences, Shahid Beheshti University of Medical Sciences, Tehran, Iran; 3 Shahid Beheshti University of Medical Sciences Faculty of Medicine, Taleghani General Hospital, Department of Cardiology, Tehran, Iran

**Keywords:** SDF-1, Monocytes, Platelets, Co-culture, Foam cells

## Abstract

**Objective:**

Far beyond hemostasis and thrombosis, significant evidence has indicated the critical role of platelets in atherosclerosis. SDF-1 is among the pro-inflammatory chemokines that are increased in platelets of patients with coronary artery disease (CAD). The goal of the current work is to identify the in vitro effect of platelets from either CAD patients or healthy volunteers on the induction of macrophages and foam cells.

**Materials and Methods:**

The expression of SDF-1 on platelet surfaces in CAD patients and healthy volunteers was investigated using flow cytometry. We also evaluated the CXCR4/CXCR7 expression on monocytes from buffy coats of healthy volunteers. The effect of platelets from CAD patients and healthy volunteers on differentiation of monocytes and foam cell formation was evaluated using Oil Red O (ORO) staining. Flow cytometry and real-time PCR were also employed to evaluate surface markers and mRNA expression of genes involved in this process after co-culture of platelets with monocytes.

**Results:**

Monocytes in co-culture with platelets acquired a spindleshape appearance and ORO-positive lipid droplets. In addition, platelets could induce CD163 expression, as an important marker of M2 macrophage, and upregulate the mRNA expression of the *SRB, CD36, ACAT, LXR-α*, and *ABCA1* genes in monocytes. Notably, platelets of CAD patients with higher expression of SDF-1, increased the expression of genes encoding SRB and CD36 as compared to platelets of healthy volunteers.

**Conclusion:**

Our results indicate that platelets from CAD patients could provoke monocyte differentiation into macrophages with an M2 phenotype, which in turn may participate in an atheroprotective process.

## Abstract (tr)

**Amaç:**

Hemostaz ve trombozun çok ötesinde, önemli kanıtlar trombositlerin aterosklerozdaki kritik rolüne işaret etmektedir. SDF-1 koroner arter hastalığı (KAH) olan hastaların trombositlerinde artan pro-enflamatuvar kemokinler arasında yer almaktadır. Bu çalışmanın amacı, KAH olan hastalar ve sağlıklı gönüllülerin trombositlerinin makrofajlar ve köpük hücrelerinin indüksiyonunda in vitro etkilerini belirlemektir.

**Gereç ve Yöntemler:**

KAH olan hastalar ve sağlıklı gönüllülerde trombosit yüzeyindeki SDF-1 ekspresyonu akım sitometri ile incelendi. Biz ayrıca sağlıklı gönüllülerin beyaz kan hücrelerinin bir tabakasından elde edilen monositlerde CXCR4/CXCR7 ekspresyonunu değerlendirdik. KAH olan hastalar ve sağlıklı gönüllülerin trombositlerinin monositler ve köpük hücreleri oluşumu üzerine etkisi Oil Red O (ORO) boyası kullanılarak değerlendirildi. Yüzey belirteçlerini ve trombositlerin monositlerle ortak kültürü sonrası süreçteki genlerin mRNA ekspresyonunu değerlendirmek için ayrıca akım sitometri ve gerçek zamanlı polimeraz zincir reaksiyonu çalışıldı.

**Bulgular:**

Trombositler ile ortak kültüre edilen monositler iğ şeklinde görünüm ve ORO-pozitif yağ damlacıkları kazandılar. Ayrıca, trombositler M2 makrofajın önemli bir belirteçi olan CD163 ekspresyonunu indüklediler ve monositlerdeki S*RB, CD36, ACAT, LXR-α*, ve *ABCA1* genlerinin mRNA ekspresyonunu up-regüle ettiler. Özellikle, SDF-1 ekspresyonu yüksek olan KAH olan hastaların trombositleri, sağlıklı gönüllülere göre SRB ve CD36’yı kodlayan genlerin ekspresyonunu artırdı.

**Sonuç:**

Bulgularımız, KAH olan hastaların trombositlerinin, monositlerin M2 fenotipinde makrofajlara farklılaşmasına neden olduklarını ve böylece ateroprotektif sürece iştirak ettiklerini göstermektedir.

## Introduction

Atherosclerosis is a multifactorial process that leads to cardiovascular diseases and involves a complex interaction between cellular and non-cellular constituents, which include platelets, cytokines, and leukocytes [1]. Far beyond hemostasis and thrombosis, the critical role of platelets in wound healing, inflammation, atherosclerosis, and the immunity system has been recognized [2,3]. Platelets store a wide variety of cytokines, chemokines, mitogens, angiogenic factors, and other bioactive molecules in their granules [4,5], which can potentially induce pro- and anti-inflammatory responses [6]. Several investigations have reported the crucial role of chemokines in the pathogenesis of cardiovascular diseases [7]. SDF-1 (CXCL12) is among the pro-inflammatory chemokines released following platelet activation [8,9]. Circulating platelets of patients with coronary artery disease (CAD) have elevated expression of SDF-1 [10,11], which is associated with cardio-protection and enhanced left ventricular functional recovery after myocardial infarction [12,13]. At injury sites, platelets recruit and activate leukocytes through cell-cell interactions or indirectly by release of their mediators [14]. Remarkably, platelet-derived SDF-1 can bind to its chemokine receptors, CXCR4 and CXCR7, on monocytes and facilitate phagocytosis of apoptotic platelets by these cells and promote their differentiation into foam cells [15].

Classically, macrophages are classified into two major subtypes: pro-inflammatory M1 macrophages and anti-inflammatory M2 macrophages [16]. Briefly, M1 macrophages sustain the inflammatory response through production of inflammatory cytokines. On the other hand, M2 macrophages contribute to wound repair and tissue regeneration [17]. According to the expression of surface markers, CD86 is used as an M1 macrophage marker [18], while CD163 is commonly used to detect M2 macrophages [19]. 

We hypothesized that increased expression of SDF-1 on the platelets of CAD patients will promote differentiation of monocytes into macrophages or foam cells. Therefore, the aim of this study was to investigate the in vitro effect of CAD patients’ platelets in induction of macrophage and foam cells in comparison with normal platelets.

## Materials and Methods

### Materials

Mouse monoclonal anti-human CD61-FITC (Dako, Denmark), mouse monoclonal anti-human CXCR4-PE (BioLegend, USA), mouse monoclonal anti-human/mouse CXCR7-PE (BioLegend, USA), mouse monoclonal anti-human SDF-1-PerCP (Novus Biologicals, USA), mouse monoclonal anti-human CD14-PE (Beckman Coulter, USA), mouse monoclonal anti-human CD11b-PE (Dako, Denmark), mouse monoclonal anti-human CD11c-FITC (Dako, Denmark), mouse monoclonal anti-human CD86-PerCP (Abcam, USA), and mouse monoclonal anti-human CD163-FITC (R&D Systems, UK) were used in our study. Ficoll-Hypaque was from Lymphodex (Inno-Train, Germany), Taq DNA Polymerase 2x Master Mix RED and Real Q Plus Master Mix Green Low ROX were procured from Ampliqon (Copenhagen, Denmark), the RevertAid First Strand cDNA Synthesis Kit was from Thermo Fisher Scientific (USA), and trypan blue, Oil Red O (ORO) stain, and TRIzol were purchased from Sigma-Aldrich (USA).

### Expression of SDF-1 on Platelets of CAD Patients and Healthy Volunteers

Ten patients with symptomatic CAD who were visited in the cardiovascular section of Taleghani Hospital (Tehran) were investigated for SDF-1 expression on the surface of platelets. CAD patients were selected with respect to clinical symptoms, myocardial ischemia markers, and ECG results. The increased level of SDF-1 was an inclusion criterion for selection of CAD patients for evaluation in co-culture, while exclusion criteria were diabetes, history of CAD in the family, hypertension, hyperlipidemia, smoking, and medication usage on admission. Ten healthy volunteers with no history of cardiovascular diseases, who were matched with the CAD patients regarding age and sex (p>0.05), were also evaluated as normal controls. Finally, three patients with high expression of this marker were selected for platelet preparation in comparison with three healthy volunteers with low expressions of SDF-1. This study was approved by the Ethics Committee of Shahid Beheshti University of Medical Sciences (IR.SBMU.RETECH.REC.1396.717) and all the participants gave informed consent in accordance with the Declaration of Helsinki. To detect the surface expression of SDF-1, diluted platelet-rich plasma (PRP) was incubated with mouse monoclonal anti-human SDF-1-PerCP and mouse monoclonal anti-human CD61-FITC or their respective isotype controls and analyzed by flow cytometer (Attune NxT; Life Technologies, USA). Platelet CD61 expression was used in the present study for identifying the platelet population. We also used mean fluorescence intensity (MFI) as a quantitative indicator for the surface expression of the indicated protein.

### Isolation of Platelets

Peripheral blood was collected in ACD-A (Acid Citrate Dextrose, solution-A) anticoagulant (1:4) from CAD patients and healthy volunteers and centrifuged at 200 g for 15 min. The prepared PRP was added to Tyrode’s HEPES buffer (HEPES - 2.5 mM, NaCl - 150 mM, KCl - 1 mM, NaHCO_3_ - 2.5 mM, NaH_2_PO_4_ - 0.36 mM, glucose - 5.5 mM, BSA - 1 mg/mL, pH 6.5) and centrifuged at 800 x g for 10 min. The platelet pellet was suspended in Tyrode’s HEPES buffer (pH 7.4; supplemented with CaCl_2_ - 1 mM, MgCl_2_ - 1 mM).

### Peripheral Blood Monocyte Preparation

Buffy coats from healthy volunteers were used for isolation of peripheral blood monocytes. Subsequently, we purified monocytes through centrifugation on a Ficoll-Hypaque density gradient (20 min, 760 x g) and adhesion to a plastic surface. Non-adherent cells were removed by gentle washing after 4 h and the remaining adherent cells (monocytes) were harvested. Viability of monocytes, as measured by trypan blue dye exclusion, was >98% immediately after their isolation. Monocyte purity was assessed using flow cytometry analysis of FSC-SSC and surface CD14 expression. The expressions of CXCR4 and CXCR7 on monocytes were also evaluated by flow cytometry.

### Monocyte and Platelet Co-culture

Monocytes were grown in RPMI-1640 medium supplemented with 100 U/mL penicillin, 100 µg/mL streptomycin, 2 mM L-glutamine, and 10% fetal calf serum in a humidified atmosphere of 5% CO_2_ at 37 °C. Isolated platelets were added to monocytes (monocyte to platelet ratio of 1:100), and half of the medium was exchanged with fresh complete medium every 2 days. During co-culture, monocytes were examined by microscopic analysis. After co-culture, platelets or floating cells were washed away and monocytes were evaluated by ORO staining, flow cytometry, and real-time PCR.

### ORO Staining for Foam Cell Evaluation

For ORO staining, the cells were stained after 7, 10, and 15 days of co-culture. After 2-3 washings with PBS, 10% formalin was used to fix the cells for 30 min, and then they were penetrated with 60% isopropanol for 5 min and stained with ORO staining for 10 min at room temperature. To remove any residual ORO stain, 60% isopropanol was used to destain wells for 15 s, and next the wells were washed 3 times with PBS. Images of foam cells were taken under 10x and 40x objectives by light microscope (Olympus) with an Optika camera (Italy). Image analysis was performed using ImageJ software.

### Flow Cytometry Assessment of Monocytes

Surface expressions of markers on differentiated macrophages were analyzed using flow cytometry following 7 days of co-culture. PBS was used to wash the cells, and with fluorescent-conjugated antibodies against CD14, CD11b, CD11c, CD86, and CD163 or their respective isotype controls, cells were labeled. After 30 min of incubation in the dark at 4 °C, cells were then fixed with 0.5% paraformaldehyde and were measured using flow cytometry. Analysis of data was performed by gating on viable cells using FSC-SSC characteristics in the monocyte/macrophage population. The percentage of each marker was determined against its respective isotype control.

### cDNA Synthesis

Total RNA was isolated from harvested monocytes following 7 days of co-culture with TRIzol according to the manufacturer’s recommendation and converted to complementary DNA (cDNA) using a cDNA synthesis kit. Quantitative real-time PCR for *ABCA1, CD36, SRB, PPARγ, SRA, ACAT1, ABCG1*, and *LXR*-α *(NR1H3)* was carried out with a Rotor Gene Q kit (QIAGEN, Germany) using Real-Time PCR Master Mix. The housekeeping gene *ABL* was also used as a control for differences in RNA concentrations. All the primers that were used in our research are given in Table 1.

### Statistical Analysis

Each experimental condition was performed in triplicate to evaluate the mean ± standard deviation (SD). All data were analyzed using Prism software (USA). To determine the difference between two groups, the unpaired two-tailed Student t-test was applied. ANOVA was applied to compare multiple data. In all cases, the minimum statistical significance was p<0.05.

## Results

### Platelets of Patients with CAD Had Increased Expression of SDF-1

**Figure 1:**
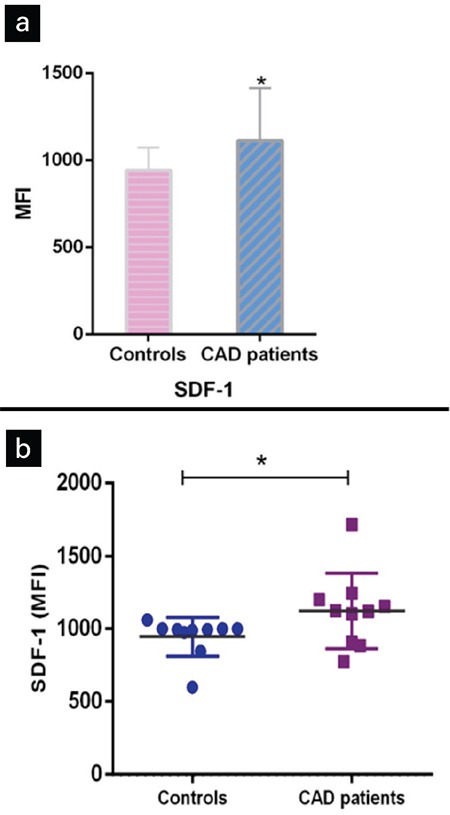
Assessment of the expression of SDF-1 on the platelets of ten healthy volunteers and ten patients with symptomatic coronary artery disease (CAD). (a) Platelet SDF-1 expression was significantly higher in CAD patients than in the healthy controls. (b) Comparison of the expression of SDF-1 on the platelets of controls and CAD patients. Statistical values are given as mean ± standard deviation. *p<0.05. MFI: Mean fluorescence intensity.

As shown in Figure 1, analysis of flow cytometry data showed that the expression of SDF-1 on the platelets of CAD patients was significantly higher than that of healthy controls (MFI: 1112±304 vs. 943±131, p=0.042).

### Surface Expression of CXCR4 and CXCR7 on Monocytes

**Figure 2:**
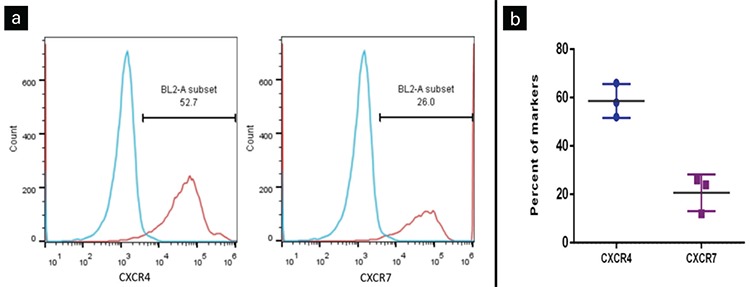
Flow cytometry analysis of CXCR4 and CXCR7 on monocytes isolated from buffy coats. (a) Histograms show that monocytes isolated from buffy coats expressed CXCR4 and CXCR7 with respect to corresponding isotype controls. (b) Comparison of expression of CXCR4 and CXCR7 on monocytes isolated from buffy coats. Experiments were conducted in triplicate.

We evaluated the expression of CXCR4 and CXCR7 on surface of monocytes which were isolated from buffy coats on the first day. Our results showed that the expression levels of CXCR4 and CXCR7 on monocytes were 51.7±3.2% and 28.5±2.5%, respectively (Figure 2).

### Platelets Significantly Induced Spindle-Shape Appearance in Macrophages

**Figure 3:**
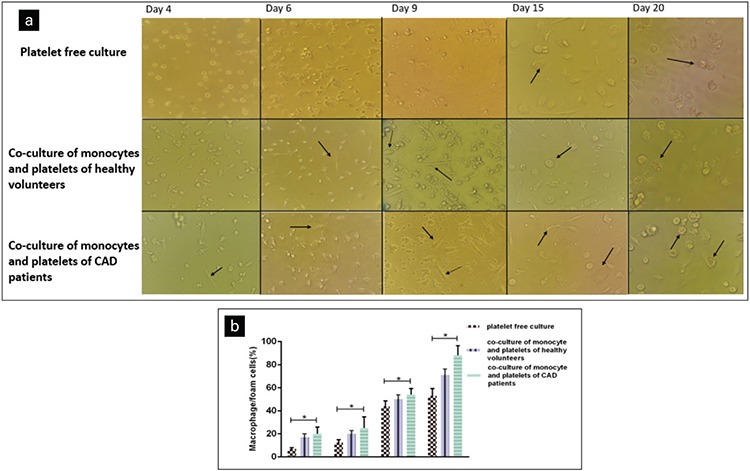
Assessment of changes in monocyte morphology at different days of culture. (a) Platelets helped monocytes differentiate into macrophages and foam cells as compared to platelet-free culture. Furthermore, platelets of coronary artery disease (CAD) patients had more monocytes differentiated into foam cells in comparison with platelets of healthy volunteers. Monocyte shape changed following co-culture with platelets as shown with arrows in the pictures. Magnification: 400x. (b) Bar diagram representing a comparison of monocyte shape changes on different days of cultures. Experiments were conducted in triplicate.

In vitro and in vivo maturation of blood monocytes into macrophages takes place as a consequence of interactions with different cell types. We evaluated monocyte morphology for 20 days of co-culture and we showed that monocyte-platelet co-culture prompted platelet clearance. Moreover, platelets induced differentiation of monocytes into macrophages with spindle-shaped appearance. After prolonged co-culture (15 days), a considerable proportion of these cells showed morphological characters of foam cells. Furthermore, our results indicated that platelets of CAD patients had enhanced differentiation of monocytes to foam cells in comparison with platelets of healthy volunteers (Figure 3).

### ORO Staining Showed Significant ORO-Stained Lipid Droplets in Monocytes

**Figure 4:**
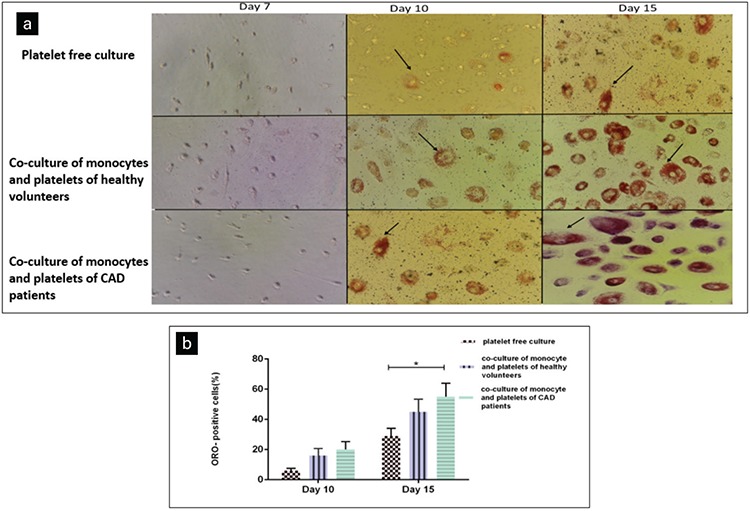
Evaluation of macrophage foam cells using Oil Red O (ORO) staining during different days of co-culture. (a) After 7 days, macrophages showed no significant ORO-stained lipid droplets, although after 10 and 15 days of co-culture, significantly more lipid droplets with ORO staining could be observed in foam cells cultured with platelets. Furthermore, ORO staining indicated that platelets of coronary artery disease patients had enhanced differentiation of monocytes to foam cells in comparison with platelets of healthy volunteers. Foam cells are shown with arrows in the picture. Magnification: 400x. (b) Bar diagram representing a comparison of OROpositive cells between different samples on days 10 and 15. Each experiment was conducted in triplicate. CAD: Coronary artery disease.

Formation of foam cells is time-dependent, so we stained and analyzed ORO-stained lipid droplets on days 7, 10, and 15 following co-culture of monocytes and platelets. After 7 days, macrophages showed no significant ORO-stained lipid droplets, but after prolonged co-culture to 10 and 15 days, lipid droplets were seen more significantly in macrophages cultured with platelets. Furthermore, ORO staining indicated that platelets of CAD patients had enhanced differentiation of monocytes into foam cells in comparison with platelets of healthy volunteers (Figure 4).

### Platelets Increased CD163 Expression on Monocytes After 7 Days

**Figure 5:**
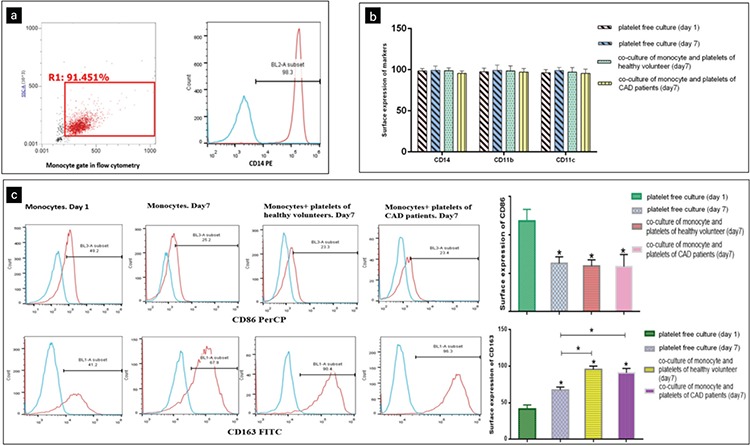
Platelets in co-culture induced monocytes to differentiate into CD163-positive macrophages. (a) Flow cytometry measurement of CD14 expression was used to assess the purity of isolated monocytes. (b) Bar graph represents phenotypes of monocytes in terms of CD14, CD11b, and CD11c surface expression in co-culture with platelets compared with platelet-free cultures. (c) Flow cytometry data represent CD86 and CD163 expressions of monocytes. Flow cytometric histograms of CD86 and CD163 expression showed substantial expression of CD163 as compared to the corresponding isotype controls on monocytes in co-culture with platelets. Representative flow cytometric bar diagrams of CD86/CD163 expression on monocytes show predominant CD163 expression of monocytes in co-culture with platelets compared with platelet-free cultures. CD86 expression of monocytes was associated with a decrease after 7 days of culture.

Monocytes express CD163 at low to moderate levels [20]; however, alternatively activated macrophages have high expressions of this marker. These cells play an important role in inhibiting the inflammatory responses and scavenging components of damaged cells [21]. Flow cytometry was conducted to detect the surface expression of monocyte markers following isolation. As shown in Figure 5a, monocyte purity, which was investigated using FSC-SSC and CD14 expression, was above 98%. Expression of other markers was analyzed on day 1 and high expressions of CD11b and CD11c and low expressions of CD163 and CD86 were identified. In monocytes on day 7, whether cultured in the presence of platelets or without platelets, high expressions of CD14, CD11b, and CD11c were detected (Figure 5b). CD86 expression on monocytes was associated with a decrease after 7 days of co-culture, independent of the presence or absence of platelets. A slightly increased expression of CD163 was observed in platelet-free cultures on day 7 compared with day 1. However, a significant increase of this marker was detected on monocytes co-cultured with platelets (Figure 5c).

### Platelets Induced the Expression of the Genes Involved in Cholesterol Absorption, Cholesterol Esterification, and Cholesterol Efflux in Monocytes

**Figure 6:**
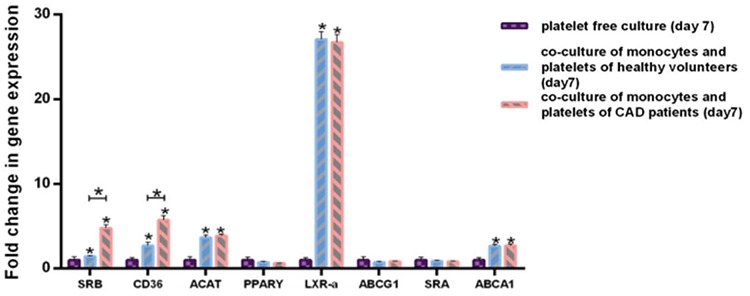
Evaluation of gene expression changes of monocytes after 7 days. After co-culture of monocytes with platelets of healthy volunteers, quantitative reverse transcription-polymerase chain reaction analysis demonstrated the increased expression of the following genes: *SRB, CD36, ACAT, LXR-α*, and *ABCA1*. Co-culture of monocytes with platelets of CAD patients had the same results, but compared to platelets of healthy volunteers, increased expression of CD36 and SRB was observed. Statistical values are given as the mean ± standard deviation of 3 independent experiments. *p≤0.05.

In this study, following co-culture of monocytes with platelets, we investigated the mRNA expression of *CD36*, scavenger receptor class A *(SRA)*, and acyl-coenzyme A cholesterol acyltransferase *(ACAT)* in monocytes, which are among the key genes in lipid accumulation [22,23]. We also evaluated the gene expression changes that are expected to enhance the cholesterol efflux from foam cells, including ATP-binding cassette transporter A1 *(ABCA1)*, ATP-binding cassette transporter G1 *(ABCG1)*, peroxisome proliferator-activated receptor γ *(PPARγ)*, liver X receptor-α *(LXR-*α*)*, and scavenger receptor class B (SRB) [24,25,26]. Our results showed that platelets elevated the expression of mRNA of *CD36, ACAT, ABCA1, SRB,* and *LXR-*α. Furthermore, platelets of CAD patients had significantly elevated mRNA expression of *CD36* and *SRB* in comparison with platelets of healthy volunteers, which indicated that these platelets play a larger role in the formation of macrophage and foam cells (Figure 6).

## Discussion

Platelets are important players in atherosclerosis and recruit other cells towards lesion sites [27]. Alpha-granules of platelets are major sources of SDF-1, a well-known chemokine that is overexpressed on the surface of platelets upon activation [28]. Notably, it has been reported that among leukocyte subtypes, monocytes preferentially interact with activated platelets through CXCR4 and CXCR7 [15]. According to studies, CD163 expression, as an important marker of M2 macrophages, is elevated in macrophages of inflamed tissues like atherosclerotic lesions [29]. Moreover, Buchacher et al. [30] indicated that monocytes with spindle-shape morphology are mainly representative of M2 macrophages. In this study, we evaluated the interaction of monocytes and platelets for 20 days. We showed that over 7 days platelets not only drive the monocytes to differentiate into spindle-shape macrophages, but also upregulate the expression of *CD163*, which is in agreement with a recent study conducted by Chatterjee et al. [15]. Our results also revealed that longer co-culture of cells resulted in a considerable proportion of macrophages presenting morphological characteristics of foam cells. Platelets of CAD patients that expressed a higher percentage of SDF-1 induced greater numbers of foam cells as compared to platelets of healthy volunteers, indicating that SDF-1 derived from platelets may play a probable role in differentiation of monocytes to foam cells.

Although previous studies discussed the contributory role of platelets in the differentiation of monocytes into foam cells [9], the precise molecular mechanisms of this effect were poorly understood. To the best of our knowledge, this is the first time that the expression levels of genes that are mainly involved in the conversion of monocytes-macrophages into foam cells were evaluated after co-culture of platelets with monocytes. Our findings demonstrated that platelets increased the mRNA expression of the *CD36*, *ACAT*,* SRB*, *LXR-*α, and *ABCA1* genes. The role of these genes per se in foam cell formation from monocytes is controversial. Multiple lines of evidence indicated that *CD36* and *ACAT* pave the way for the formation of foam cells through enhancing the capability of the cells to uptake platelets/ox-LDL and cholesterol esterification, respectively [22,23]. On the other hand, increased expression levels of *SRB*, *LXR*-α, and *ABCA1* were also reported to be associated with the cholesterol efflux from macrophage foam cells [24,25,26]. Other studies also showed that the gene expression of proteins such as *CD36*, *ACAT1*, *LXR-*α, and *ABCA1* are increased during macrophage-derived foam cell formation [22,23,31,32]. Tsukamoto et al. [33] suggested a fundamental role for *CD36* as well as *SRB*, but not *SRA*, in the formation of foam cells during ox-LDL treatment of THP-1 cells [33]. Of particular interest, co-culture of monocytes with the platelets of CAD patients resulted in a superior upregulation of *CD36* and *SRB* mRNA expression as compared with the results of co-culture using platelets of healthy volunteers.

## Conclusion

Using an in vitro co-culture of monocytes and platelets, our study indicates that platelets from CAD patients could provoke monocyte differentiation into macrophages with an M2 phenotype, which in turn may participate in an atheroprotective process.

## Figures and Tables

**Table 1:**
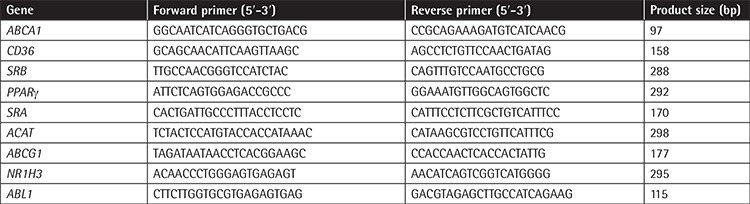
Primers that were used for cDNA synthesis.
